# Evolving modalities of atrial fibrillation ablation: From thermal to nonthermal

**DOI:** 10.1016/j.hroo.2025.12.026

**Published:** 2026-01-13

**Authors:** Margaret Harvey, Jennifer Walker, Sunil K. Jha

**Affiliations:** 1 Department of Acute and Tertiary Care, College of Nursing, University of Tennessee Health Science Center, Memphis, Tennessee; 2 Cardiac Electrophysiology, University of North Carolina Health, Chapel Hill, North Carolina; 3 Department of Medicine, College of Medicine, University of Tennessee Health Science Center, Memphis, Tennessee

**Keywords:** Atrial fibrillation, Pulse field ablation, Radiofrequency ablation, Cryoablation, Irreversible electroporation

## Abstract

This review topic provides an overview of a novel technique for catheter ablation in patients with atrial fibrillation (AF) called pulsed field ablation. Pulsed field ablation uses a nonthermal vs thermal approach (radiofrequency or cryoablation) for obtaining myocardial cell death, which is referred to as irreversible electroporation for the treatment of AF via pulmonary vein isolation. This emerging technology delivers microsecond high-voltage electrical fields that may limit damage to tissues outside the myocardium and offers a promising approach to managing AF with shorter procedural times and fewer major complications. This review aimed to educate allied health professionals regarding this evolving technology by summarizing the biophysics of irreversible electroporation, clinical evidence supporting pulse field ablation, associated complications, and future applications.

**Key Findings**

▪ Pulsed field ablation (PFA) uses a nonthermal vs thermal approach (radiofrequency ablation [RFA] or cryoablation) for obtaining myocardial cell death, which is referred to as irreversible electroporation treatment of atrial fibrillation.
▪ Electroporation is the process whereby an electrical field of sufficient energy disrupts the transmembrane resting potential, leading to increased cell membrane permeability and the formation of aqueous pores in its lipid bilayer. The external electrical energy field disrupts the cell transmembrane resting voltage, causing membrane structural and molecular changes that lead to increased cell membrane permeability.
▪ One of the main advantages of PFA is its selectivity for cardiac tissue, lowering risks of collateral damage, for example, esophageal injury, phrenic nerve injury, and pulmonary vein (PV) stenosis. Some known risks associated with PFA, although low, include coronary vasospasms, hemolysis, and vasovagal response.
▪ Clinical studies suggest that PFA is a safe, alternative nonthermal approach to inducing myocardial cell death during PV isolation catheter ablation for atrial fibrillation.
▪ Some industry partners are now advancing catheter-based technologies that combine both RFA and PFA capabilities in a single, versatile system (Affera, Medtronic).

## Introduction

Current atrial fibrillation (AF) management guidelines indicate that catheter ablation (CA) is a class 1A recommendation to improve symptoms and rhythm control in symptomatic patients, particularly when drugs fail, as first-line therapy in selected paroxysmal AF (PAF) cases.^1^ Pulmonary vein isolation (PVI) is the cornerstone of AF CA unless another trigger is identified.^1^ Despite radiofrequency ablation (RFA) or cryoablation being effective, PVI reconnections still occur in approximately 20% of patients.^2^ Thermal methods also risk complications such as atrioesophageal fistula and phrenic nerve injury.^3^^,^^4^ A nonthermal alternative, pulsed field ablation (PFA), selectively targets cardiac cells, reducing collateral damage.^5^ PFA delivers microsecond bursts of high-energy voltage via specialized catheters, inducing cell death through irreversible electroporation (IRE). This review summarizes the biophysics of IRE, clinical evidence supporting PFA, its complications, and future applications.

## IRE

**Figure 1:**
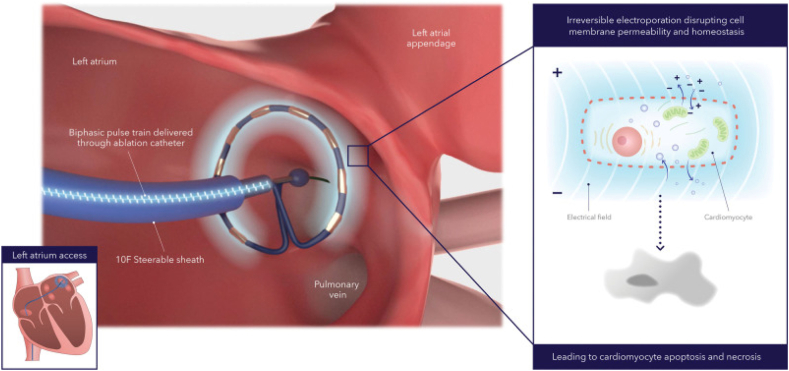
Catheter ablation method with a pulsed field ablation system. Alternating positive and negative electrodes sustain a bipolar electrical field around the catheter that extends into the tissue. The electrical field increases cell membrane permeabilization, which then leads to cell function disruption and eventually cell death (ie, apoptosis and necrosis). Reproduced from Verma et al.^14^

Electroporation is the process whereby an electrical field of sufficient energy disrupts the transmembrane resting potential, leading to increased cell membrane permeability and the formation of aqueous pores in its lipid bilayer.^6^^,^^7^ The external electrical energy field disrupts the cell transmembrane resting voltage, causing membrane structural and molecular changes that lead to increased cell membrane permeability. Reversible electroporation causes a temporary increase in cell membrane permeability, allowing for the delivery of nucleic acids, for example, DNA, into a variety of cells—such as bacteria, yeasts, plant cells, and mammalian cell lines—with wide application occurring in the fields of biomedicine, biotechnology, food science and technology, and environmental science.^6^, ^7^, ^8^ In contrast, IRE results in cell death from apoptosis, necroptosis, and pyroptosis, given that there is no window for cellular membrane repair or return of homeostasis.^6^ IRE is used when cell death is desired, for example, soft tissue tumors, or in the case of AF, in which PVI is used to target lesions and induce cell death using PFA (Figure 1).

### Preclinical trials

A systematic review of preclinical studies was conducted with healthy animal models where IRE was obtained using high-energy pulses to the ventricular myocardium, atrial tissue/pulmonary veins (PVs), coronary arteries, esophagus, phrenic nerve, and cardiac ganglia.^9^ Of the 16 studies included in the review, 320 ablations were performed using various high-voltage energy sources, for example, LIFEPAK defibrillator, and 14 various modified RFA catheter types, given that no commercial PFA-specific catheters were available. Although ablation of cardiac tissue using IRE was found to be effective without the risk of PV stenosis or arterial, nerve, or esophageal damage, significant variation occurred in pulse duration, repetition, and amount of energy delivered among all studies, with recommendations that terms and reporting criteria for IRE be standardized.^9^ The first-in-human study reported successful PVI (100%) using pulsed electrical fields in all 15 patients with AF.^10^ The ablation was performed using a customized over-the-wire endocardial catheter for percutaneous transseptal PVI using a mean of 3.26 ± 0.5 lesions/PV and a total PFA energy delivery time of <60 seconds per patient with no reported complications, thus establishing the efficacy of tissue-specific, ultrarapid ablation for AF.^10^ The feasibility and safety of single-pulse IRE PVI using a multielectrode impedance system were evaluated in 10 patients with AF. All PVs were successfully isolated with a mean of 2.4 ± 0.4 IRE applications per PV with a mean delivered peak voltage of 2154 ± 59 V and peak current of 33.9 ± 1.6 A, with no periprocedural complications being observed, thus indicating that acute bidirectional electrical PVI could be achieved safely by single-pulse IRE ablation.^11^^,^^12^

## Clinical studies

The PULSED AF pilot trial, a nonrandomized, prospective, multicenter, global, premarket clinical study, was designed to test the feasibility and efficacy of the PulseSelect PFA system (Medtronic) using a bipolar, biphasic electrical field through a circular multielectrode array catheter. Of the 38 patients enrolled, PFA was achieved in 100% of the patients, with no serious adverse effects observed during the 30-day follow-up.^13^ In 2023, the US Food and Drug Administration (FDA) approved the PulseSelect PFA system for CA of patients with PAF and persistent AF. The 12-month effectiveness and safety of the PulseSelect PFA system were evaluated in the PULSED AF pivotal trial, a prospective, global, multicenter, nonrandomized, paired single-arm study of patients with symptomatic AF refractory to class 1 or 3 antiarrhythmic agents. The PFA catheter was positioned at each PV ostium and assessed using fluoroscopy or intracardiac echocardiography imaging. The Medtronic PulseSelect PFA system was used to deliver biphasic, bipolar waveforms to an over-the-wire, circular array with 9 gold electrodes. 1 application consisted of 4 biphasic, bipolar pulse trains, each lasting 100–200 ms at 1400–1500 V. The catheter was rotated circumferentially after each application to a new position to achieve full circumferential isolation. Of the 150 patients with PAF and 150 with persistent AF, 66.2% and 55.1%, respectively, had effective PFA for 1 year with a low rate of primary safety adverse events (0.7%).^14^

1-year clinical outcomes using PFA for the treatment of PAF were evaluated in 3 multicenter studies (ie, IMPULSE, PEFCAT, and PEFCATII) using the Farapulse endocardial ablation system with a basket or flower PFA over-the-wire catheter. In 121 patients, PVI was achieved in 100% of PVs with PFA alone, and the 1-year Kaplan-Meier estimate for freedom from any atrial arrhythmias (AAs) was 84.5% ± 5.4%, thus demonstrating that the PFA catheter provided excellent durability and safety for successful PVIs.^15^ The ADVENT trial evaluated the efficacy and safety of PFA using the Farapulse pentaspline PFA CA system (Boston Scientific, Inc) vs conventional thermal ablation for PVI in a randomized, single-blind, noninferiority trial for patients with drug-refractory PAF.^16^ Each PV received 2 applications in a partially open “basket” configuration, followed by rotation and subsequent sets of 2 more applications for a total of 8 PFA applications per PV. The primary efficacy end points of no events occurring (freedom from PVI procedural failure, atrial tachycardia lasting >30 seconds, use of class 1 or 3 antiarrhythmic agents or cardioversion after the 3-month blanking period, and repeat ablation within 1 year) were obtained in 73.3 % of the 304 patients assigned to the PFA group compared with the 71.3% of the 302 assigned to the thermal ablation group. The incidence of primary safety endpoints was 2.1% in the PFA group vs 1.5% in the conventional group with an overall posterior probability of noninferiority, that is, >0.999, concluding that the ADVENT trial demonstrated that PFA was noninferior for safety and treatment success.^17^ Secondary analysis from the ADVENT trial examined the impact of postablation AA burden on outcomes and the effect of ablation modality on AA burden using weekly transtelephonic electrocardiograms and 72-hour Holter recordings at 6 and 12 months. An AA burden of >0.1% was associated with poorer quality of life and increased interventions such as redo ablations. Results showed that, compared with thermal ablation, PFA had a less clinically significant threshold of 0.1% AA burden.^18^ In 2024, the FDA approved the Farapulse PFA ablation system (Farapulse, Inc) for PVI drug-refractory, recurrent, symptomatic PAF.

The Assessment of Safety and Effectiveness in Treatment Management of Atrial Fibrillation With the Biosense Webster Irreversible Electroporation Ablation System study was a multicenter, single-arm study evaluating the long-term safety and efficacy of an integrated PFA and 3-dimensional mapping system for patients with drug-refractory PAF.^19^ Across 30 centers, 277 patients with PAF underwent PFA with at least 12 PVI applications per PV. All patients treated with the PFA catheter achieved acute procedural success (100%). 12-month freedom from documented atrial tachyarrhythmia was 75.4%. The primary adverse event rate was 2.9% (8 of 272), with the most common complication being pericardial tamponade (1.1%), followed by major vascular access/bleeding (0.7%), pericarditis (0.4%), stroke (0.7%), and transient ischemic attack (0.4%). The primary adverse event rate was similar between those patients treated without fluoroscopy (1 of 69; 1.4% [95% confidence interval 0–7.8]) and >0 minutes of fluoroscopy (7 of 203; 3.4% [95% confidence interval 1.4–7.0]; *P* = .68). The investigators concluded the safety and effectiveness of the variable-loop PFA catheter, with short procedure and PFA application times and low fluoroscopy exposure and results being similar to the insPIRE study that used and integrated PFA and a mapping system.^19^^,^^20^ VARIPULSE (Johnson & Johnson MedTech) received FDA approval for use of the PFA system for the treatment of drug-refractory PAF on November 7, 2024.

## Complications

**Figure 2:**
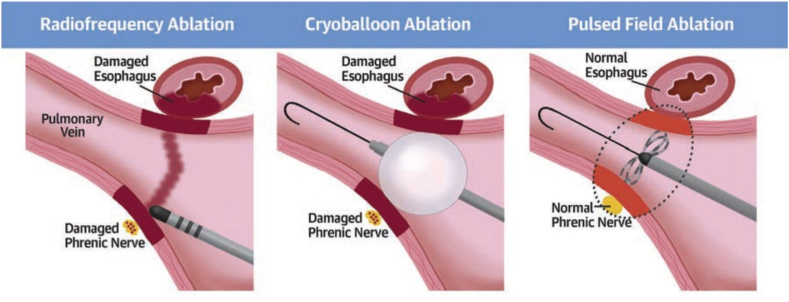
A comparison of safety concerns for pulmonary vein isolation using thermal vs nonthermal approaches. Adapted from Reddy et al.^39^

One of the main advantages of PFA is its selectivity for cardiac tissue, lowering risks of collateral damage, for example, esophageal injury, phrenic nerve injury, and PV stenosis (Figure 2). A retrospective study of 1237 AF ablations compared the incidence of complications between PFA (n = 156) and RFA (n = 316).^21^ Pericardial effusion was higher with RFA than with PFA (71.4% vs 34.0%; *P* < .001). However, hemolysis (9.0% vs 0%), coronary events (5.8% vs 0.6%), and vasovagal responses (14.1% vs 0%) were higher with PFA than RFA (*P* < .001). Esophageal damage (0% vs 4.1%; *P* < .001) and PV stenosis (0% vs 1.9%; *P* = .184) were only seen with RFA, there were fewer deaths with PFA (2.6% vs 8.9%; *P* = .010), and ischemic stroke rates were similar (5.8% for PFA vs 6.3% for RFA; *P* = .805).^21^

### Coronary vasospasm

Coronary vasospasm observed during PFA is a class effect and may be mediated by activation of the sympathetic nervous system, electrically induced contraction of vascular smooth muscle cells, or ion imbalance from IRE.^22^ Owing to anatomic proximity, vasospasm of the left circumflex artery may occur during mitral isthmus ablation, and right coronary vasospasm during cavotricuspid isthmus (CTI) ablation. Severity ranges from transient spasm to ST-elevation with ventricular arrhythmias, although most cases are reversible.^21^ In a retrospective analysis of 25 patients undergoing PFA (pentaspline catheter) for AF, the effects of PFA lesion sets and nitroglycerin (NTG) on coronary vasospasm were evaluated. During PVI and left atrial posterior wall ablation, coronary spasm did not occur, but severe subtotal coronary vasospasm during CTI ablation occurred in 5 of 5 consecutive patients (100%) and was relieved by intracoronary NTG.^23^ Another study found that pretreatment with NTG decreased the frequency and severity of coronary vasospasm during CTI ablation.^24^ Although NTG may be effective when administered prophylactically or for the acute treatment of coronary vasospasm, further studies are needed to determine the optimal protocol.

### Hemolysis

Hemolysis is a complication seen more frequently with PFA than thermal ablation. With PFA, IRE induces red blood cell damage, leading to the release of hemoglobin into the circulation (hemolysis). Excess intravascular hemoglobin can be nephrotoxic by causing oxidative tubular injury, obstruction from pigment casts, and activation of inflammatory cascades, resulting in acute kidney injury (AKI).^25^ Risk factors for AKI with PFA include older age, chronic kidney disease, diabetes mellitus, hypertension, structural heart disease, depressed left ventricular ejection fraction, and a higher number of PFA applications.^26^ The incidence, severity, and clinical impact of PFA-induced hemolysis were investigated in a multicenter observational study of 145 patients undergoing AF CA with a pentaspline PFA catheter (biphasic, bipolar pulses of 2 kV) and 70 patients receiving RFA (40–90 W) at 4 European centers.^25^ Ablation was performed using the Farastar PFA generator (Farapulse) with multiple PFA deliveries, each consisting of 5 biphasic, bipolar pulses of 200 ms duration and 300 ms interpulse pauses with 2 kV output energy. For PVI, a minimum of 4 deliveries were applied in the basket configuration, followed by a minimum of 4 deliveries in the flower configuration for each PV with slight catheter rotation before applying the following 2 deliveries. PFA was associated with increased intravascular hemolysis (94.3% vs 6.8%; *P* < .001) compared with RFA. Free plasma hemoglobin, lactate dehydrogenase, and bilirubin levels were higher after PFA, and haptoglobin levels were lower. The severity of hemolysis was associated with an increased number of PFA applications. Although no cases of significant anemia were observed between groups, there were 4 cases of stage 1 AKI (3.2%) in the PFA group compared with 0 cases in the RFA group (0%; *P* = .305).^25^

Strategies to reduce hemolysis and the risk of AKI may include minimizing the number of PFA applications and instituting hydration protocols. A study of 103 patients with AF undergoing PFA evaluated the effect of postablation hydration (0.9% sodium chloride ≥2 L) on renal function.^27^ In the group without postablation hydration (n = 28), serum creatinine at 24 hours was significantly higher than baseline (1.46 ± 0.28 mg/dL vs 0.86 ± 0.24 mg/dL; *P* < .001), and in 4 cases, the serum creatinine increased above 2.5 mg/dL. There were no significant changes in serum creatinine in the group that received postablation hydration (n = 75).^27^ Thus, careful consideration of risk factors for AKI, hydration, and close monitoring of renal function is important to minimize the occurrence of AKI after PFA.

### Vasovagal response

Vasovagal response is one of the most common complications reported in PFA, typically during left superior PV ablation owing to stimulation of the ganglionated plexus (GP).^21^ Del Monte et al^28^ tested the hypothesis that PFA-induced transient anterior-right GP modulation, when targeting the right superior PV before any other PVs, may effectively prevent intraprocedural vagal responses. In a prospective cohort study of 80 patients, vasovagal reactions were significantly lower when ablation began at the right superior PV (13% vs 78%; *P* < .001). This strategy temporarily modulates the anterior-right GP, suppressing the parasympathetic response.^28^ Temporary pacing or atropine can also be used to manage vasovagal responses during PFA.^21^^,^^28^

### Silent cerebral events

The incidence of periprocedural stroke in patients undergoing AF ablation is approximately less than 1%.^29^ In contrast, the incidence of silent cerebral events (SCEs) is higher and ranges from 2.5% to 54.5%.^30^ SCEs may occur during thermal or nonthermal AF ablation owing to various factors such as air or thrombus via sheaths, clot formation on the catheter or ablation lesion, or gas bubble formation. Intraprocedural cardioversion, prolonged procedural time, persistent AF, and spontaneous echocardiographic contrast on transesophageal echocardiography are associated with SCEs.^31^ Hu et al^30^ conducted a systematic review and meta-analysis including 86 trials with 10,456 patients undergoing AF ablation to evaluate the incidence of SCEs across different energy modalities. The overall incidence of SCEs was 19.1%, with no significant difference occurring between PFA and thermal ablation.

SCEs may result from microembolic signals (MESs), but the incidence of MESs during PFA for AF remains unknown. Shiomi et al^32^ sought to determine the incidence of MESs in 33 patients undergoing PFA and compared the number of MESs observed during procedures performed on each of 3 PFA systems. Real-time vascular parameters were monitored throughout the procedure using carotid echocardiography, and 1 day after the procedure, cerebral magnetic resonance imaging was performed. PVI was performed at the ostium and antrum, according to the recommended number of energy applications. Investigators found that the number of MESs was significantly lower in Farapulse than in PulseSelect (*P* = .01) and VARIPULSE (*P* < .001) and that multiple SCEs were exclusively observed with VARIPULSE. In March 2025, the company issued corrections to the instructions for use after reports of higher-than-expected periprocedural stroke/transient ischemic attack rates. The advisory specifically calls for using VARIPULSE only in patient populations and conditions included in the clinical studies (not off-label expansion) and procedural adjustments such as ensuring adequate hydration and avoiding lesion stacking to reduce thromboembolic risk.^33^

## Future PFA applications

Studies using PFA technology for other arrhythmias and approaches are ongoing. The ADVANTAGE AF study is a prospective, single-arm, multicenter investigational device exemption study to assess the safety and efficacy of PVI plus posterior wall ablation for patients with persistent AF using the Farapulse PFA system. In 339 patients, the success for both PVI and posterior wall ablation was 97%, with a primary safety endpoint of 2.3% and a primary effectiveness of 63.5% at 1 year, suggesting favorable safety and outcome.^34^ The feasibility and safety of mitral isthmus ablation in addition to PVI and posterior wall ablation with PFA were studied in patients with persistent AF using a PFA catheter (Farawave, Farapulse). Of the 45 patients enrolled, complete mitral isthmus block was achieved in all with a 20% recurrence rate during a mean follow-up time of 107.8 ± 59.5 days.^35^ The safety, effectiveness, and procedural outcomes of PFA in treating atrial flutter (AFL) were evaluated in 311 consecutive patients undergoing AFLs using the Farawave PFA catheter with guided intracardiac echocardiography. All AFL circuits were successfully ablated with 96.5% acute procedural success, 99.5% success for CTI-dependent flutters, and 85.4% success for perimitral circuits. AFL recurrence over a median follow-up of 175 days was 3.9%, demonstrating that PFA for AFL ablations was safe and effective.^36^ A systematic review evaluating the outcomes of PFA for ventricular tachycardia included a total of 6 studies (5 case reports and 1 case series) where a variety of cardiac mapping and ablation delivery systems were used. In 5 studies, dedicated PFA generators, catheters, and sheaths manufactured by Farapulse (Farastar, Faradrive, Farawave) were used, with 1 study using the CENTAURI pulsed field system by Galaxy Medical. 88% of the procedures were successful with 100% long-term efficacy and no procedural complications.^37^ In a randomized, single-blind, noninferiority trial, 420 patients with persistent AF underwent ablation using a large-tip catheter with dual pulsed field and RF energies vs ablation using a conventional RFA system. The primary effectiveness endpoint was observed in 73.8% and 65.8% of patients in the investigational and control arms, respectively (*P* < .0001 for noninferiority), with shorter procedural times in the investigational arm than the control arm (*P* < .0001). In October 2024, Medtronic received US FDA approval for the Affera mapping and ablation system with the Sphere-9 catheter, an all-in-one electrophysiology offering capable of PFA and RFA to treat persistent AF and CTI-dependent AFL.^38^

## Conclusion

PFA uses a nonthermal approach for inducing myocardial cell death called IRE during PVI for the treatment of AF. This emerging technology delivers microsecond high-voltage electrical fields that may limit damage to tissues outside the myocardium and offers a promising approach to the management of AF with shorter procedural times and fewer adverse events. Although some minor adverse events exist, these can be avoided by reducing the number of PFA applications. In summary, PFA is a burgeoning field within cardiac electrophysiology. Some industry partners are now advancing catheter-based technologies that combine both RFA and PFA capabilities in a single, versatile system (Affera, Medtronic). This dual-energy approach allows physicians to select the optimal ablation modality for each patient and specific cardiac tissue, reflecting ongoing efforts toward more refined, effective, and safer treatments for arrhythmias such as AF.

## Disclosures

The authors have no conflicts of interest to disclose.
